# Role and use of evidence in health system response to COVID-19 in Nigeria: a mixed method study

**DOI:** 10.11604/pamj.2023.44.191.38990

**Published:** 2023-04-20

**Authors:** Chinyere Ojiugo Mbachu, Adanma Chidinma Ekenna, Uju Patricia Agbawodikeizu, Obinna Onwujekwe

**Affiliations:** 1 Health Policy Research Group, University of Nigeria, Enugu Campus, Enugu, Nigeria,; 2 Department of Community Medicine, University of Nigeria Teaching Hospital, Enugu, Nigeria,; 3 Department of Social Work, Faculty of Social Sciences, University of Nigeria, Nsukka, Nigeria,; 4 Department of Health Administration and Management, University of Nigeria, Enugu Campus, Enugu, Nigeria

**Keywords:** COVID-19, decision-making, evidence, health systems, health system response, disease preparedness, pandemics, laboratories, disease outbreaks, hygiene, Nigeria, humans, government agencies, communicable disease control, COVID-19 diagnostic testing

## Abstract

**Introduction:**

evidence-based decision-making in health is an aspiration needed to effectively respond to current outbreaks and prepare for future occurrences. This paper examines the roles and use of evidence in health systems response to COVID-19 in Nigeria.

**Methods:**

this was a mixed method study comprising nine key informant interviews and rapid review of 126 official online documents, journal articles and media reports published from December 2019 to December 2020 with a national and sub-national focus. Key informants were drawn from the government agencies that were involved in making or implementing decisions on the health sector response to COVID-19. Data collection was performed by three researchers. Thematic analysis and narrative synthesis of data was done.

**Results:**

various forms of evidence were used to make decisions on Nigeria´s health system response to COVID-19, and these are broadly classified into three, namely, i) lessons learned from past experiences such as community engagement activities, early recognition of risks and deployment of non-pharmaceutical pandemic control measures, ii) proven interventions with contextual relevance like the emphasis on hand hygiene education for health workers, and iii) risk assessment and situation analysis reports like adopting a multi-sector response to COVID-19 control, expanding COVID-19 diagnostic laboratories to new sites across the country, and relax lockdown restrictions while maintaining key limitations to curb a spike in COVID-19 cases.

**Conclusion:**

Nigeria´s health system response to COVID-19 upheld the use of evidence in making critical decisions on the prevention and control of the pandemic.

## Introduction

Nigeria has had a fair share of disease outbreaks in the past decade, requiring concerted efforts by government and non-government agencies in emergency response. During the Ebola outbreak in 2013, 20 cases and 8 deaths were recorded in Nigeria [1]. Lassa fever was declared an emergency in 2019 due to high case fatality rates [2]. The most recent yellow fever outbreak which affected five States in Nigeria claimed 172 lives [3]. Since these outbreaks have been mostly confined (that is not of a global nature) health systems responses have been localized. For instance, a country-specific incident management system was activated in Nigeria during the 2013 Ebola outbreak to ensure timely data collation, analysis, and dissemination to stakeholders [1]. This strategy was needed because the national health information system was weak. Contrariwise, the health system response to COVID-19 outbreak took on a global/international approach because the disease defied national and continental boundaries. The COVID-19 pandemic created a conundrum of shocks to the global health infrastructure, with low resource settings being worse off [4,5]. This necessitated the reeling out of protocols and guidelines by apex international health organisations, and the adoption or adaptation of these protocols and guidelines by member States/countries. The foremost protocols and guidelines for COVID-19 were developed with the “speed of lightning” to ensure that the response to a growing epidemic was prompt and timely enough to halt the spread of the virus [6-8]. For that reason, countries were urged and prodded to quickly adopt and begin to implement globally recommended strategies, even if contextual appropriateness had not been determined.

Global recommendations do not always have universal application [9,10]. Therefore, when copied “verbatim” and implemented at national or subnational levels without recourse to contextual realities, globally recommended strategies may not achieve desired health outcomes. Several instances of where efficacious interventions have failed to achieve health improvements have been recorded in literature [11-15]. In almost every case, the failures were attributed to poor adaptation of strategies resulting from a lack of contextual understanding [11,14,15]. Irrespective of the need for countries to act fast in their response to COVID-19, indigenous actions ought to scrutinize global strategies and make them fit for context. In Nigeria, as in other countries affected by the pandemic, national and subnational governments (and non-government agencies) are constantly making decisions and taking actions to improve the overall health system response to COVID-19. The way and manner in which evidence is used in making decisions could predict the outcomes of such decisions in terms of effectiveness, efficiency, and sustainability. Evidence-based decision-making in health is an aspiration, more so when it comes to managing disease outbreaks. Evidence is needed to effectively respond to current outbreaks and prepare for future occurrences. This would typically include information from published research studies, programme and policy evaluations, and historical data [16]. A few studies have reported what constitutes evidence use in policy making for health programmes [17,18]. However, literature on evidence-based decision-making during disease outbreaks (particularly pandemic situations) is grossly lacking. This paper examines the roles and use of evidence in health systems response to COVID-19 in Nigeria. It highlights the types of evidence that were used, the decisions they influenced, and how these sources of evidence were generated and disseminated. This information is particularly useful to policymakers and programme managers in low and middle-income countries who may be faced with health emergency situations where health systems are weak, health resources are limited, and adoption of global recommendations to local settings need to be driven by evidence to ensure efficient resource utilization. The findings from the review could guide in the selection of types/sources of evidence to be used in making specific decisions.

## Methods

We adopted a mixed method (parallel and convergent) study design that comprised key informant interviews (KIIs) and scoping review of official (government and non-government) documents, journal articles and media reports published from December 2019 to December 2020. The geographical scope was national and sub-national. Ethical approval was obtained from the health research ethical committee of the authors´ institution.

### Key informant interviews

Nine (9) key informants were purposively selected from the various units of the coordinating committee in the COVID-19 response at the Federal and State levels, including from the Nigerian Centre for Disease Control, National Primary Health Care Development Agency, National Health Insurance Scheme, State Emergency Operations Centres and State Primary Health Care Development Agencies. The KIIs were conducted using an interview guide that explored the role of evidence in decision-making for health systems response to COVID-19.

The interviews were conducted by three of the authors who have experience in qualitative research methods. Written consent was obtained from participants before the interview guide was administered. All interviews were audio recorded with the consent of the participants.

Audio files of KIIs were transcribed verbatim. Initial codes were developed by reading a couple of rich transcripts. The initial coding framework was tested on a random selection of two transcripts and the final coding framework (Table 1) was then applied to all the transcripts. The coding of transcripts was done in NVivo software according to the themes and subthemes in the coding framework. Data coded under each thematic area were retrieved by running coding queries, and the outputs of each query were summarized into narratives and the findings were organized according to the three major forms of evidence that were used to make decisions for health systems response to COVID-19 in Nigeria.

**Table 1:** coding framework for examining the roles and use of evidence in decision-making for health systems response to COVID-19 in Nigeria

Code	Code definition
a)Describe decision maker	Descriptive information about the interviewee
a.ii) Describe decision organization	Descriptive information about the interviewee’s organization
b)HS processes description	General description of the processes that were institutionalized during the COVID-19 pandemic with respect to the building blocks. This includes the names of the processes and when they were implemented
c)Decisions types	The types of decisions that were made in the implementation of HS response for COVID-19. This includes policy decisions, funding decisions, executive decisions, operational decisions.
c.ii) Decisions structures	Description of the explicit efforts that were made or established (or that were leveraged) for decision-making for the HS response that were implemented. Examples include EOC, TWG, COVID-19 research coalition
c.iii) Decisions rationale	What were the reasons for making the decisions about the HS response to implement and how this should be done? Example, the decision to phase the roll-out of COVID-19 vaccination campaign was based on occupational risk exposure
c.iv) Decisions evidence	What types of evidence were used to make the decisions about the HS response that were implemented; what was evidence sourced; how was evidence used?
d)Actors roles, interests and relationships	Who was involved in the decision-making for the HS response that were implemented for COVID-19; what roles did these actors play, who did they work with and what were their interests?
d.ii) Actors influence	External influence (or lack of it) on the actors
Emerging themes	A code for flagging any information that you are unsure how to code

**Document review:** Research articles were sourced from online journals written in English language and published from December 2019 to December 2020. Search terms/queries were generated using various combinations of keywords that represent six (6) categories of interest (Table 2). Primary search terms comprised keywords selected from the primary keywords (column 2) while secondary search terms comprised combinations of secondary keywords (column 3). The search was conducted in English and performed in PubMed, Google Scholar and Scopus. Published official documents were retrieved from organizations´ websites. The documents included policies, strategies, plans, protocols, guidelines, minutes of expert meetings, situation reports, and expert recommendations to government, written in English language.

**Table 2:** primary and secondary keywords used to create search items

Search category	Primary search keywords	Secondary search keywords
Disease	COVID-19	Coronavirus, COVID, pandemic
Health sector	Health system CACOVID	Health sector, government, private sector, community response,
Epidemic containment measures	Non-pharmaceutical measures Diagnostic, testing and isolation	Face mask, hand hygiene, sanitizer, lockdown, social/physical distancing Community testing, door-to-door campaign
Healthcare ecosystem	Physical infrastructure Drugs and medical equipment Workforce Data infrastructure Financing	Health facility, laboratories Supplies, consumables Health workers, human resources for health HMIS, health information systems, DHIS Funding, finance, donations, grants
Other sectors	Environment, information, food & agriculture, finance, education, aviation, internal affairs, industries	Immigration
Coordination	Task Force NCDC	

Media reports were retrieved online from mainstream national dailies (Vanguard, Punch, Guardian, The Nation, Business Day, and Premium Times) that report national and State level responses to COVID-19. Media search was performed in FACTIVA to ensure consistency in the review. We also reviewed other online news sources that have consistently reported health systems response to COVID-19 in Nigeria. Some of these are Nigeria Health Watch, TalkNaija, Devex, PWC Nigeria, APO Africa Newsroom, COVID-19 Nigeria by Innover, and Wella Health Triage. The search terms were applied to the headlines and lead paragraphs of media articles.

The review was performed by three reviewers. A repository of all documents and media reports was generated and independent reviewers were assigned documents and media reports to read and extract data. Each article/document was read by one reviewer, and relevant data were extracted verbatim into a uniform data extraction template that was designed for this study. The template was structured into themes and subthemes that capture processes of decision-making, stakeholders involved and use of evidence.

The data extracted by each reviewer were synthesized across documents and media reports for each thematic area of the review to generate a comprehensive transcript of findings for each thematic area. The thematic transcript from the document review was summarized through narrative synthesis by two reviewers and the summary reports were merged.

## Results

In this section, we highlight the types of evidence used, and the decisions that were informed by the evidence. Where possible, we also mention how the evidence was generated and disseminated.

**Figure 1:**
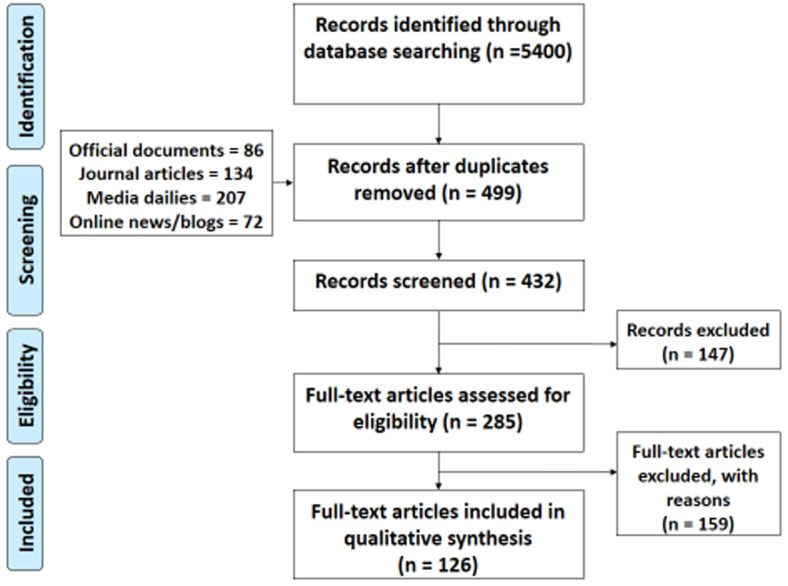
PRISMA diagram for the scoping review

The PRISMA flow chart shows the numbers of documents retrieved and included in the review (Figure 1). A total of 5400 records were identified from various sources including PubMed (895), Google Scholar (1233), Scopus (25), Factiva (2951), organizational websites (103), online news and blogs (193). After duplicates were removed and titles were scanned, 432 records remained. A further 147 records were excluded because the full texts could not be retrieved online, and 159 articles were excluded for not meeting the eligibility criteria. Thus, 126 full-text articles were reviewed and included in the qualitative synthesis.

Various forms of evidence were used to make decisions on Nigeria´s health system response to COVID-19, and these are broadly classified into three, namely, i) lessons learned from past experiences, ii) proven interventions with contextual relevance, and iii) risk assessment and situation analysis reports (Table 3). Explicit reference to the use of evidence in decision-making was found in only 13 of the documents that were included in the review.

**Table 3:** evidence-informed decision-making for COVID-19 health sector response in Nigeria

Types or forms of evidence	Evidence-informed decisions that were made
Lessons learned from polio eradication initiative and control of previous epidemics	Provided guidance for planning community engagement activities to enhance response and preparedness for COVID-19 at the community levels FG’s establishment of screening at ports of entry, intensification of media sensitization, and free testing of symptomatic patients Early recognition of risks and deployment of non-pharmaceutical pandemic control measures were based on past experiences and successes in epidemic control
Proven interventions/strategies	S-I-N approach was adopted for early recognition and source control for IPC in hospital settings WHO’s “My 5 moments for hand hygiene” is being used to train health workers and educate communities on the steps in hand hygiene Distribution of soaps in IDP camps as part of the hand washing campaigns Engineering and environmental controls such as adequate ventilation, physical distancing and environmental hygiene
Risk assessment & Situation analysis reports	Constitution of a COVID-19 mitigation team was based on an assessment of high risk of importation and local transmission of infection in the country Decision to adopt a multi-sectoral response for the effective containment of COVID-19 was based on recognitions of a weak Nigerian health system Expansion of COVID-19 diagnostic laboratories by NCDC was based on establishment of community transmission trend Decision to relax the lockdown restrictions (while maintaining key limitations to curb a spike in COVID-19 cases) was based on evidence indicative of emerging epidemic control An increase in reporting of sexual violence within one week of the nationwide lockdown necessitated the decision by a non-government agency to violate the lockdown and continue to offer health services to victims of sexual assault Workable solutions for continuing cancer care during the COVID-19 pandemic were based on considerations of resource limitations and lack of clinical protocols

### Lessons learned from the polio eradication initiative and controlling previous epidemics

Owing to its previous successes in controlling epidemics, Nigeria was one of the first countries to recognize the risk of COVID-19 and deploy non-pharmaceutical measures to control community spread and limit importation of new cases [19]. The community response and preparedness plan for COVID-19 was informed by lessons learnt from polio eradication initiative in Nigeria which is that engaging communities through their leaders and gatekeepers plays a critical role in ensuring high rate of compliance with interventions and enhances community ownership of the process of communication interventions [20]. Hence, the community response and preparedness plan clearly stipulate that stakeholder engagement activities which will be implemented at community levels will be steered by community leaders and gatekeepers. Also, building on lessons learned from the 2014 Ebola response, the Federal government responded by establishing screening procedures at ports of entry, amplifying media sensitization, and making testing free for symptomatic patients [21].

### Proven interventions/strategies & contextual relevance

Findings from documents and KIIs confirmed that context-relevant ‘evidence’ from proven interventions and best practices informed the COVID-19 response in Nigeria. Although efforts were made to contextualize global recommendations, these guidelines determined the direction of Nigeria´s response to COVID-19. “We used [evidence] based on our context; based on what we think that can work for us. Then, global [evidence]; we cannot dissociate ourselves from what is the best practice´ (Male, State Incident manager) “…we wanted to make sure that whatever we were doing was based on available evidence and that we were doing what was globally accepted as at that time” (Female, National decision maker).

Regarding vaccination campaign, the key informants noted that evidence of proven effectiveness influenced the adoption of COVID-19 vaccination and national and subnational levels. “When vaccines also became available and all that and there was evidence that the vaccines were working, we also were able to convince both the senate, the government, and the executive that Nigeria needed this vaccine; and they were brought in. They were able to put mechanisms in place to make sure that we push these vaccines also to the [State and local government] levels” (Female, National decision maker). The Screen, Isolate and Notify (S-I-N) approach was adopted for early recognition and source control for IPC in health facilities or hospital settings [22]. Secondly, the WHO´s ‘My 5 moments for hand hygiene’ was used to outline the steps in hand hygiene in the training curriculum of PHC workers and for demonstration during community awareness events [22].

In the IDP camps, sensitization campaigns on hand washing were accompanied with distribution of soaps to camp dwellers because this was consistent with findings that have established the relationship between the distributions of soaps in humanitarian settings with increase in practice of hand washing [19]. Furthermore, the inclusion/use of engineering and environmental controls such as adequate ventilation and environmental cleaning/hygiene in IPC was based on recent studies that suggest that COVID-19 could be airborne [23]. Hence, the control and prevention of COVID-19 transmission requires the control of air flow with use of specially designed ventilation systems in addition to standard precautions. Regarding considerations for surgical procedures, the country´s IPC plan stipulates that the risks and benefits of surgery for a COVID-19 patient needing it should be weighed because *recent data point to a high proportion of post-operative pulmonary complications associated with increased mortality in patients with COVID-19* [22].

### Risk assessment & Situation analysis reports/findings

Decision about what strategies to adopt in the COVID-19 response were informed by reports of ongoing risk assessment and situation analysis. These kinds of information were gathered through the review of empirical evidence, expert opinions of technical working groups, and verbal reports from community representatives. “There is a technical working group that will agree on the way to go. It was not just one person; and like I told you the presidential task force also had a lot of experts. And we were getting opinion from across the world” (Female, National decision maker).

Soon after Nigeria recorded its first (imported) and second (index) cases of COVID-19, the Federal government constituted a COVID-19 mitigation team. Additional risk assessment considerations in constituting the team were the weak state of the health system and the fact that WHO had named Nigeria as *“one of the probable hotspots for the transmission of COVID-19 in the African continent”* [24].

Federal government´s decision to adopt a multi-sectoral response for the effective containment of COVID-19 was based on the realization that the pandemic had shaken the foundations of seeming stable health systems around the world [25]. The expansion of COVID-19 testing sites beyond the existing diagnostic laboratories that were equipped with the requisite PCR machine in the country was based on a trend of community transmission. An excerpt from the report by Adesanya (2020) reads: *“As evidence of community transmission emerged, the need to expand testing capacity further in order to gain an accurate picture of case incidence figures became imperative, leading the NCDC to publish a national strategy for the expansion of COVID-19 testing capacity”* [26].

The decision to adopt facility- or home-based management strategies were based on the limited availability of resources to care for the increasing number of infected people. As the available health facilities (including bedspace, personnel, and supplies) became overwhelmed by new cases of COVID-19, Lagos State government adopted home-based management for mild and asymptomatic cases and expanded their capacity through public-private partnerships with accredited private hospitals and laboratories. “[We] expanded to include the private facility. We, the government, were of the opinion that we could have some sanity, because for much of the first phase and part of the second phase, we had issues of crowd control. The health centres were inundated with people crowding the health facility” (Male, State program officer).

The decision to relax the lockdown restrictions (while maintaining key limitations to curb a spike in cases) during the third phase of the country´s response to COVID-19 was based on reduction in test positivity ratio to less than 5% (which is indicative of emerging epidemic control) and a progressive fall in case fatality rate to 1.9% [23]. The evidence was reviewed by the PTF and a recommendation to ease lockdown restrictions was made.

Similarly, the decision by Mirable Centre (a sexual assault referral centre) to continue to provide health services to victims of sexual assault irrespective of the nationwide lockdown was based on evidence of an increase in reporting of sexual violence following mandatory lockdown [27].

The workable solutions developed by Nigerian oncology experts for continuing cancer care during the COVID-19 pandemic were based on considerations of infrastructural and resource limitations for cancer care in the country, as well as the absence of clear guidelines for managing cancer patients during the pandemic [28].

## Discussion

Our research underscores that Nigeria´s health system response to COVID-19 upheld the use of evidence in making critical decisions on the prevention and control of the pandemic. When the MEACOC was inaugurated, they were charged to use scientific expertise and necessary evidence to ensure that the health sector response to COVID-19 results in a flattening of the epidemic curve [29]. Moreover, during the third phase of easing of lockdown restrictions the use of data and science to inform decision-making was identified as a major priority of the country´s response to COVID-19 [23]. Hence, various forms of evidence were used to decide and define COVID-19 control strategies that are being implemented through the health system in Nigeria.

Some of the strides that Nigeria has made in the fight against COVID-19 can be attributed to lessons learned from controlling previous epidemics (specifically Ebola and polio), and the ongoing epidemic of Lassa fever [19-21]. In recognition of the risks associated with the pandemic, the government of Nigeria rapidly instituted activities and measures taken to control transmission, reduce morbidity and mortality; protect and care for vulnerable populations. Hence, many decisions that were taken by government and non-government agencies in response to the COVID-19 pandemic were influenced by risk assessment reports, the situation of COVID-19 in the country, and the resultant health and social consequences. Decisions on strategies to adopt to prevent and control COVID-19 infection in Nigeria were based on evidence of proven effectiveness and ability to adopt/adapt in the Nigerian context. The country´s response strategy is comparable to what is being implemented in other sub-Saharan countries, and hinges on non-pharmaceutical interventions such as physical/social distancing, personal hygiene and isolation of confirmed cases [30].

Evidence for decision-making in disease response can be drawn from many sources. Lessons are drawn from many sectors and in some areas in particular in disease outbreaks, such as zoonotic surveillance activities, support of medical and public health systems to improve local and international responses to epidemics, risk communication, the role of social media in outbreak awareness, and response, production of better diagnostic tools, creation of better therapeutic medications, and design of better vaccines [31]. On the other hand, it can be argued that the use of evidence is not an easy feat depending on the local context [32]. This is because the union of science and public administration is a complex system in public health preparedness and disease response [32-34]. Our findings in the types of evidence from risk assessment and situation analysis/reports demonstrates how the local context can elicit a peculiar response, for instance, services to victims of sexual assault. In contrast, the local government of China in early 2020 shunned scientific evidence and expert´s advice but rather focused on avoiding blame in spite of prevailing local evidence [32]. This stance fuelled the spread of the SARS-CoV-2 globally.

The use of situational analysis as evidence in the COVID-19 response was not peculiar to Nigeria. A situational report of the importation of the disease into Ghana on the 22^nd^ of March 2020 necessitated the policy of travel restrictions (air, land and sea) to limit disease importation [35]. A similar scenario played out in Rwanda. After an initial 30-day ban on travel and restrictions on social gatherings, the continued rise in the number of COVID-19 cases led to the government issuance of sweeping infection control measures to promote social distancing in the country [36]. Leveraging on already existing structures of proven interventions or strategies, the government of Rwanda strengthened its mental health services which it had made widespread in its health system, in the course of the pandemic [37]. Researchers in Sierra Leone drew a parallel between the Ebola Virus Disease (EVD) and the COVID-19 and stated that lessons concerning social responses (reducing contact with patient and minimizing burial risks, for instance) to EVD provided a useful starting point for COVID-19 [38].

The importance of the role of evidence in disease response is perhaps that globalization fosters local compliance to guidelines [39]. A view from South Africa makes a distinction between the Bantu and Khoikhoi tribes and how the values of the Khoikhoi tribe in entitlements according to age opposed the country´s vaccination plan that prioritized essential workers, the elderly and other vulnerable groups [40]. The Khoikhoi tribe would prioritize the young for health care response in the pandemic, access to ventilators and vaccines, over the elderly [40]. This disparity had the potential to fuel societal non-compliance to COVID-19 vaccination. Therefore, application of global guidelines should be adopted and adapted in view of local contexts for desired results.

Health emergency preparedness includes all actions undertaken in anticipation of a likely emergency. These actions cut across myriad activities, from policy and legislation, public awareness, data collation, and analysis. As disease outbreaks occur in the African region, lessons are drawn from many sectors to improve local and international responses to epidemics [31]. This study happens to be the first to document evidence-based decision-making in the context of a global pandemic. However, it is limited in the use of information from published reports alone. The validity of the findings could be strengthened by triangulating with the perspectives of decision makers, and this should be explored in future.

## Conclusion

Findings from our exploration of the use of evidence in the COVID-19 response in Nigeria clearly demonstrate that proven strategies and interventions, lessons from previous epidemics and the polio legacy strengthened the country´s preparedness and response to the COVID-19 pandemic. With the intent of controlling spread of the virus, Nigeria adopted global strategies which were adapted to suit local realities as situation reports kept evolving. This adaptation in the decision-making increases societal compliance to preventive measures and yields desired containment of the COVID-19 pandemic.

### What is known about this topic

- *Efficient disease outbreak response stem from evidence-based knowledge used by decision-makers and relevant global and local stakeholders;*
- *Evidence becomes more important when dealing with novel public health issues or diseases;*
- *Even when available, policy makers may not employ evidence-based interventions or may adopt global standards unsuitable in their local contexts*.

### What this study adds

- *Although the document review found explicit reference to evidence-based decision-making in a very small fraction (approximately 10%) of documents that were included in the review, Nigeria´s health system response to COVID-19 upheld the use of evidence in making critical decisions on the prevention and control of the pandemic;*
- *Our findings clearly demonstrated that proven strategies and interventions, lessons from previous epidemics and the polio legacy strengthened the country´s preparedness and response to the COVID-19 pandemic*.
